# Clinical Manifestations of Punta Toro Virus Species Complex Infections, Panama, 2009

**DOI:** 10.3201/eid2305.161925

**Published:** 2017-05

**Authors:** Nathan D. Gundacker, Jean-Paul Carrera, Marlene Castillo, Yamilka Díaz, Jose Valenzuela, Ashutosh Tamhane, Brechla Moreno, Juan Miguel Pascale, Robert B. Tesh, Sandra López-Vergès

**Affiliations:** University of Alabama at Birmingham, Birmingham, Alabama, USA (N.D. Gundacker, A. Tamhane);; Gorgas Memorial Institute for Health Studies, Panama City, Panama (J.-P. Carrera, M. Castillo, Y. Díaz, J. Valenzuela, B. Moreno, J.M. Pascale, S. López-Vergès);; University of Texas Medical Branch, Galveston, Texas, USA (R.B. Tesh).

**Keywords:** Punta Toro virus, Punta Toro virus infection, viruses, clinical manifestations, Panama, vector-borne infections, sand fly fever group, phlebovirus, Bunyaviridae

## Abstract

An investigation in Panama found that Punta Toro virus species complex (PTVs) may contribute to febrile illnesses with symptoms mirroring those of dengue fever. However, further studies are needed to determine if PTV infection causes only a mild disease or if it can have more serious manifestations in some patients.

Acute febrile illness in the New World tropics has a broad differential diagnosis largely dependent on locale and seasonal outbreaks. In Central America, most febrile illnesses have historically been attributed to dengue or malaria. However, recent evidence from Panama suggests varied differential diagnoses, including hantavirus, chikungunya virus, and Zika virus infection (*1*,*2*). In 2009, a dengue outbreak was reported in Panama City, Panama. The Gorgas Memorial Institute in Panama City tested dengue-negative samples from this outbreak for alphaviruses, flaviviruses, and phleboviruses and detected Punta Toro virus species complex (PTVs) in some samples. PTV (genus *Phlebovirus*, family *Bunyaviridae*), a member of the sand fly fever group, was initially described in humans in 1966 after being isolated from a soldier in Panama who had fever, headache, myalgia, and leukopenia (*3*). The phylogenetics of PTV have been thoroughly characterized (*4*–*6*), but our search of the literature did not reveal reports of other PTV cases in humans.

The symptoms of sand fly–associated phlebovirus infection vary, but most infections cause a mild febrile illness characterized by retroorbital headache, weakness, back pain, and leukopenia. However, infection with 2 other phleboviruses, mosquitoborne Rift Valley fever virus and tick-associated severe fever with thrombocytopenia syndrome virus, causes severe disease. Little is known regarding the signs, symptoms, and clinical course of PTV infection in humans.

During the 2009 investigation, the Gorgas Memorial Institute analyzed 4,852 samples from persons in Panama with suspected acute dengue; 1,667 (34.4%) of the samples were dengue-negative. We further analyzed 201 of these samples for phlebovirus (Technical Appendix Table 1). In brief, we extracted viral RNA from the samples and evaluated it by using *Phlebovirus* genus–specific reverse transcription PCR (RT-PCR) based on the highly conserved L (large) gene that detects Toscana, Naples, Sicilian, Aguacate, Punta Toro, and Rift Valley fever viruses (*7*). We also screened samples using panflavivirus and panalphavirus RT-PCRs. 

Of the 201 samples, 27 (13.4%) were RT-PCR–positive for phlebovirus. BLAST (https://blast.ncbi.nlm.nih.gov/Blast.cgi) nucleotide sequence comparison suggested all were PTVs; 1 was previously described as Cocle virus (*4*). We conducted phylogenetic analyses on 11 of the phlebovirus-positive samples, using a 482-nt sequence and MEGA7 software (*8*). An optimal maximum-likelihood tree confirmed the samples were PTVs; all samples from 2009 (GenBank accession nos. KY43355–KY435365) clustered together close to Cocle virus (Technical Appendix Table 2) (*4*). Our attempts to isolate virus were unsuccessful. To determine if PTV had been previously detected, we tested 202 randomly selected dengue virus–negative samples from 2008; none was phlebovirus-positive.

Clinical data sheets were available for 92.6% (25/27) of the PTV-positive samples. After de-identifying the data, we entered it into a dataset. A control group consisted of 90 dengue virus–positive patients from 2009 who were frequency matched by age and randomly selected from available records. The PTV-positive case-patients were largely located in the Panama City metropolitan area (Figure). Case-patients and controls were compared primarily with regard to reported symptoms (Technical Appendix Table 3). Case-patients were significantly less likely than controls to have exanthema (22% vs. 54%; odds ratio 0.23, 95% CI 0.08–0.66; p = 0.01). We found no major clinical differences between case-patients and controls with regard to other symptoms. No patients in either group had shock or hemorrhagic syndrome.

**Figure:**
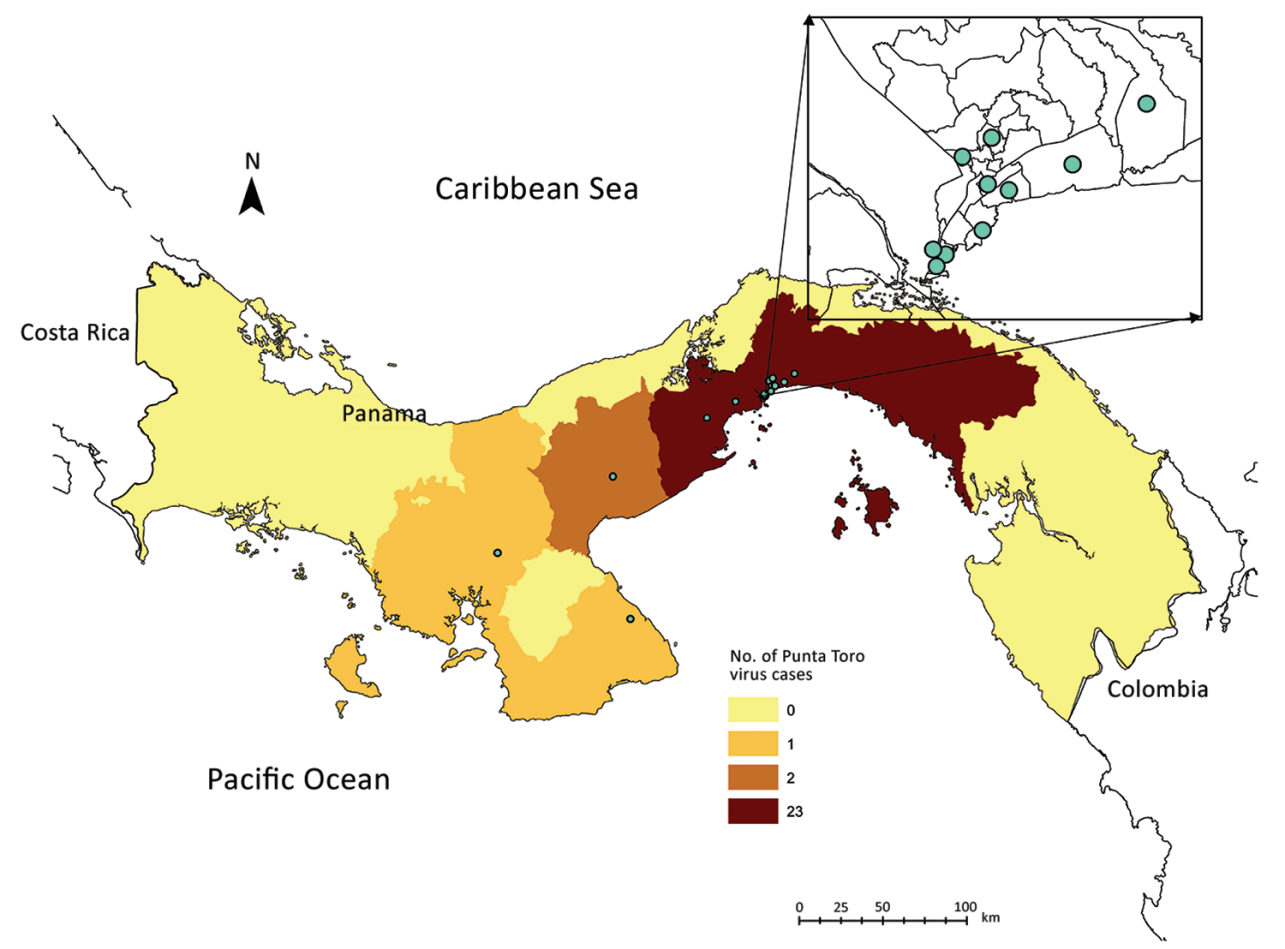
Distribution of confirmed Punta Toro species complex infections, Panama, 2009. Dots indicate cases. Inset shows enlargement of Panama City area.

Febrile syndromes in the tropics are often treated empirically; clinical decisions are often made on the basis of epidemiologic information and concurrent outbreaks. In Central America, dengue fever and malaria are treated without confirmatory testing because testing is costly and time-consuming. However, an increasing number of agents responsible for causing febrile illnesses have been identified in recent years. The variety of clinical outcomes observed with hantavirus and dengue, chikungunya, and Zika virus infections underscores the need for more accurate diagnostics to differentiate between causative agents. Clinical decisions must rely on accurate diagnoses because symptomatology is not an accurate predictor of the true etiology of a febrile illness.

Our findings suggest that, in Panama, PTVs may be a major contributor to febrile illnesses with symptoms mirroring those of dengue fever. However, the clinical course and range of disease caused by PTVs are unknown. Prospective studies are needed to determine if PTV infection causes only mild disease or if it can have serious manifestations in some patients. 

PTVs are assumed to be sand fly–borne, and sand flies are usually present in rural or forested areas (*9*). However, most cases of PTVs infection in Panama in 2009 were in urban and periurban areas, raising questions about the vector, the vector’s habitat, and the mode of virus transmission. Panama City, however, is home to two thirds of the country’s population and has improved healthcare infrastructure, which may explain the higher number of confirmatory tests from Panama City versus other areas of Panama and might result in a sampling bias. Despite these limitations, the recent Zika outbreak has shown the speed at which vectorborne diseases can spread and highlights the importance of detecting emerging viruses like PTVs.

Technical Appendix Phylogenetic tree of Punta Toro species complex (PTVs), location and number of dengue-negatives samples in Panama analyzed for PTVs in 2009, and demographic characteristics and symptoms of persons with PTV infection versus those with dengue.
