# Supplementary material for: Clinical Manifestations of Punta Toro Virus Species Complex Infections, Panama, 2009
Source: Emerg Infect Dis. 2017 May;23(5):872–4. doi: 10.3201/eid2305.161925 (PMC5403033; doi:10.3201/eid2305.161925)
Supplement: Technical Appendix — Phylogenetic tree of Punta Toro species complex (PTVs), location and number of dengue-negatives samples in Panama analyzed for PTVs in 2009, and demographic characteristics and symptoms of persons with PTV infection versus those with dengue. [file 16-1925-Techapp-s1.pdf]

Article DOI: https://dx.doi.org/10.3201/eid2305.161925

Clinical Manifestations of Punta Toro Virus Species Complex Infections, Panama, 2009

Technical Appendix

Technical Appendix Table 1. Location and number of dengue negative samples analyzed for Punta Toro species complex, 2009

Provinces of Panama	DENV negative samples	Number analyzed for Phlebovirus	Percent analyzed per province	Number positive for PTV
Bocas del Toro*	0	0	–	–
Chiriqui	53	6	11.3	0
Cocle	129	13	10.1	2
Colon	12	2	16.7	0
Darien†	15	0	0.0	–
Herrera	17	2	11.8	0
Kuna Yala	5	1	20.0	0
Los Santos	44	7	15.9	1
Ngobe Bugle‡	1	0	0.0	–
Panama Este	31	3	9.7	0
Panama Metro	691	99	14.3	18
Panama Oeste	172	17	9.9	3
San Miguelito	433	44	10.2	2
Veraguas	53	6	11.3	1
Total	1667	201	12.1	27‡

*No samples received from Bocas del Toro Region.
†Samples from Darien and Ngobe Bugle were lost before analysis.
‡From the 27 samples positive for Phlevorius, one was Cocle virus previously described (*1*), and the other 26 are described for the first time.

Technical Appendix Table 2. Comparison of demographics and symptoms between Punta Toro species complex and Dengue virus patients*

Characteristic	Overall N (%)	PTV (N = 25) n (%)	DENV (N = 90) n (%)	Odds Ratio (95% CI)†	p-value†
Demographics					
Age (years) ‡	24 (13–37)	24 (16–37)	22 (12–37)	–	–
Sex					
M	49 (45)	13 (52)	36 (42)	–	–
F	61 (55)	12 (48)	49 (58)	–	–
Symptoms					
Fever	112 (97)	25 (100)	87 (97)	2.04 (0.19–277.74)	0.65
Chills	105 (91)	23 (92)	82 (91)	1.12 (0.22–5.65)	0.89
Severe Headache	104 (90)	22 (88)	82 (91)	0.72 (0.18–2.92)	0.64
Retro-orbital pain	83 (72)	17 (68)	66 (73)	0.77 (0.30–2.02)	0.60
Myalgia	94 (82)	21 (84)	73 (81)	1.22 (0.37–4.03)	0.74
Arthralgia	81 (70)	15 (60)	66 (73)	0.55 (0.22–1.38)	0.20
**Exanthem**	**52 (45)**	**5 (20)**	**47 (52)**	**0.23 (0.08–0.66)**	**0.01**
Cough	24 (21)	8 (32)	16 (18)	2.18 (0.80–5.91)	0.13
Sore Throat	22 (19)	4 (16)	18 (20)	0.76 (0.23–2.50)	0.65
Coryza	13 (11)	3 (12)	10 (11)	1.09 (0.28–4.31)	0.90
Hepatomegaly	3 (3)	0 (0)	3 (3)	0.49 (0.004–5.30)	0.65
Splenomegaly	1 (1)	0 (0)	1 (1)	1.17 (0.01–22.62)	0.93
Nausea/vomiting	49 (43)	9 (36)	40 (44)	0.70 (0.28–1.76)	0.45
Diarrhea	23 (20)	3 (12)	20 (22)	0.48 (0.13–1.76)	0.27
Petechiae	7 (6)	1 (4)	6 (7)	0.58 (0.07–5.08)	0.63

*Bold font indicates the only symptom that was statistically significantly difference between PTV and dengue. CI = Confidence Interval; DENV = Dengue virus; PTV = Punta Toro species complex; Missing data: Age (PTV = 1, DENV = 6); Sex (DENV = 5).
†Calculated using unconditional logistic regression method. The estimates are calculated using maximum likelihood method except for fever, hepatomegaly, and splenomegaly where Firth's method was used due to zero cell frequency (*2*).
‡Age reported as median with quartiles.

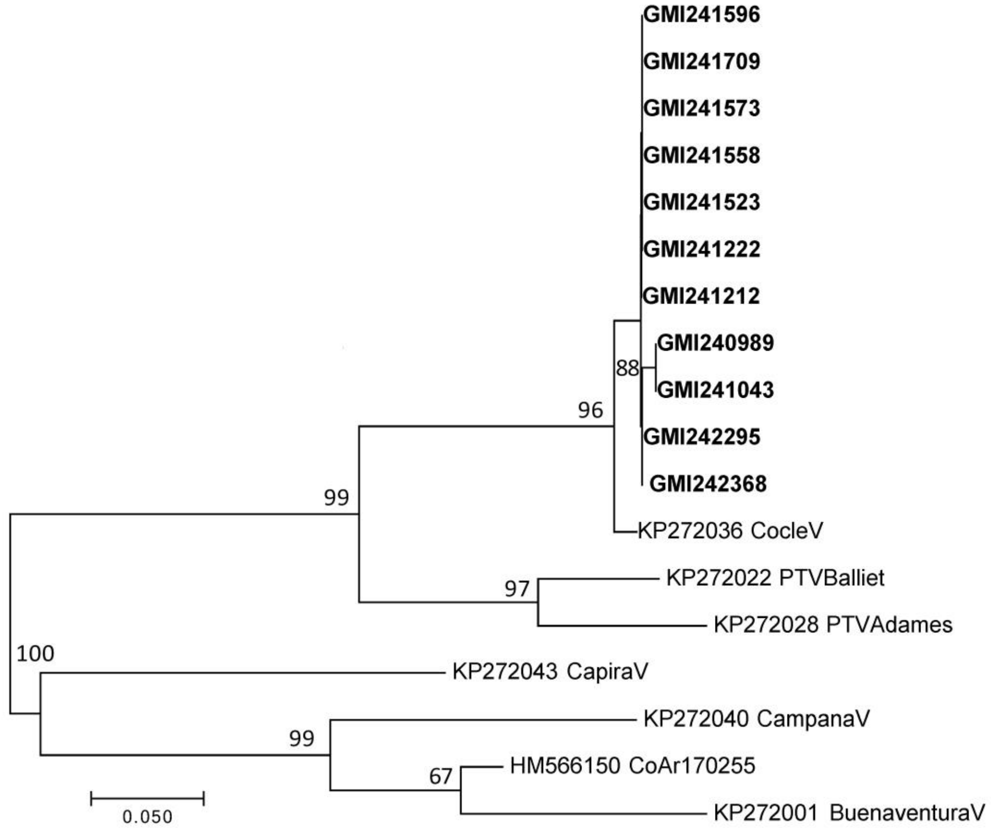

Technical Appendix Figure. Maximum likelihood phylogenetic tree of the Punta Toro serocomplex sequences detected in dengue-negative samples of febrile patients in Panama from 2009. Maximum Likelihood method based on the Tamura-Nei model. The tree with the highest log likelihood is shown, with branch lengths measured in the number of substitutions per site. The analysis involved 18 nt sequences of around (482 bp nucleotide long - L gene from 2096 to 2577) of which 11 were from 2009 Panamanian sequences (GMI240989 [GenBank accession no. KY435355], GMI241043 [accession no. KY435356], GMI241212 [accession no. KY435357], GMI24122 [accession no. KY435358], GMI241523 [accession no. KY435359], GMI241558 [accession no. KY435360], GMI241573 [accession no. KY435361], GMI241596 [accession no. KY435362], GMI241709 [accession no. KY435363], GMI242295 [accession no. KY435364], GMI242368 [accession no. KY435365]). The tree reliability topology was estimated using bootstrap resampling (2,000 replicates), bootstrap values are shown for each branch. Buenaventura virus was used as an outgroup. Evolutionary analyses were conducted in MEGA7 (*3*). From the 11 sequences, 7 are identical, whereas 2 (GMI241212 and 241222) have one mutation (bootstrap 0) and 2 (GMI240989 and 241043) have three mutations and form an inside cluster (bootstrap 88), they cluster separately from Cocle virus (11 mutations from the other 2009 Panama PTV, bootstrap 96).

References

1. Palacios G, Wiley MR, Travassos da Rosa AP, Guzman H, Quiroz E, Savji N, et al. Characterization of the Punta Toro species complex (genus *Phlebovirus*, family *Bunyaviridae*). J Gen Virol. 2015;96:2079–85. PubMed http://dx.doi.org/10.1099/vir.0.000170

2. Fisher Box J. Guinness, Gosset, Fisher, and Small Samples. Stat Sci. 1987;1:45–52 (cited 2016 Oct 31). http://projecteuclid.org/euclid.ss/1177013437

3. Kumar S, Stecher G, Tamura K. MEGA7: Molecular Evolutionary Genetics Analysis version 7.0 for bigger datasets. Mol Biol Evol. 2016;33:1870–4. PubMed http://dx.doi.org/10.1093/molbev/msw054
